# A matter of time and proportion: the availability of phosphorus-rich phytoplankton influences growth and behavior of copepod nauplii

**DOI:** 10.1093/plankt/fbaa037

**Published:** 2020-08-27

**Authors:** Cédric L Meunier, Emily M Herstoff, Carla Geisen, Maarten Boersma

**Affiliations:** ALFRED-WEGENER-INSTITUT HELMHOLTZ-ZENTRUM FüR POLAR- UND MEERESFORSCHUNG, BIOLOGISCHE ANSTALT HELGOLAND, POSTFACH 180, 27483 HELGOLAND, Germany; DEPARTMENT OF ECOLOGY & EVOLUTION, STONY BROOK UNIVERSITY, 650 LIFE SCIENCES BUILDING, STONY BROOK, NY 11794-5245 USA; ALFRED-WEGENER-INSTITUT HELMHOLTZ-ZENTRUM FüR POLAR- UND MEERESFORSCHUNG, BIOLOGISCHE ANSTALT HELGOLAND, POSTFACH 180, 27483 HELGOLAND, Germany; ALFRED-WEGENER-INSTITUT HELMHOLTZ-ZENTRUM FüR POLAR- UND MEERESFORSCHUNG, BIOLOGISCHE ANSTALT HELGOLAND, POSTFACH 180, 27483 HELGOLAND, Germany; University of Bremen, Naturwissenschaften 2, Leobener Straβe, 28359 Bremen, Germany

**Keywords:** phytoplankton quality, selective feeding, ecological stoichiometry, food web, zooplankton

## Abstract

Although consumers may use selective feeding to cope with suboptimal resource quality, little work has examined the mechanisms that underlie selective feeding, the efficiency of this behavior or its influence on consumer growth rate. Furthermore, a consumer’s exposure to suboptimal resources may also influence the consumer’s behavior and life history, including growth rate. Here, we studied how the availability of P-rich and P-poor phytoplankton influences the growth and behavior of copepod nauplii. We observed that copepod nauplii preferentially feed on P-rich prey. We also found that even relatively short exposure to P-rich phytoplankton yielded higher nauplii growth rates, whereas the presence of P-poor phytoplankton in a mixture impaired growth. Overall, we observed that swimming speed decreased with increasing phytoplankton P-content, which is a behavioral adjustment that may improve utilization of heterogeneously distributed high-quality food in the field. Based on our results, we propose that the optimal prey C: P ratio for copepod nauplii is very narrow, and that deviations from this optimum have severe negative consequences for growth.

## INTRODUCTION

High-quality food is not always available in pelagic environments, and, when it is, it may be patchily distributed, or mixed with food items of lower quality (Wiborg 1976; Andersen et al. 2009). Phytoplankton elemental content, and its quality as food for herbivores, is directly influenced by the abiotic environment (Persson *et al.,* 2010; Sterner and Elser, 2002). Fluctuations in light and nutrient availability lead to variations in microalgal content of carbon (C), nitrogen (N) and phosphorus (P), as well as the ratio of these elements (Sterner and Elser, 2002). In contrast to phytoplankton, most zooplankton species are much less variable in their body composition; thus, only a small range of food nutrient stoichiometry is of optimal quality for grazing zooplankton (Frost *et al.,* 2006). Although consumers are exposed to poor-quality diets for substantial periods, the effect of exposure time to poor quality diets and recovery potential in herbivores has not been intensively studied. The few studies on this topic have identified that the duration of the exposure to low- and high-P food significantly influences zooplankton growth. For instance, studying the freshwater cladoceran *Daphnia magna,*Becker and Boersma (2005) found that when the availability of P was decreased to every other day, zooplankton growth decreased significantly compared to animals continuously fed enriched phytoplankton. Increasing this period lowered zooplankton growth slightly more (Becker and Boersma, 2005). In contrast, also for *D. magna,*Sterner and Schwalbach (2001) showed that, during alternating P phytoplankton quality conditions, juveniles could compensate for periods of low P. This compensation suggests that daphnids can reallocate stored compounds for growth, at least over short intervals. In the only study that we are aware of using marine copepods (i.e. *Acartia tonsa*), Malzahn and Boersma (2012) showed that even though developmental rates returned back to normal after a short period of feeding P-limited algae, there was no compensatory growth, and those animals with a P-limited history always lagged behind those that were on a full-P diet the whole time. One of the major differences between copepods and daphnids is their feeding mode. Where daphnids are filter-feeders and highly unselective, copepods display a strong selectivity, and because of the array of behaviors copepods can adopt to deal with suboptimal phytoplankton quality, copepods should react differently depending on whether they encounter phytoplankton patches of homogeneous or heterogeneous quality. When high- and low-quality phytoplankton are mixed, copepods do select for high-quality phytoplankton and probably only suffer when the skewness in the mixture toward low-quality food is too strong. Conversely, when copepods are exposed to prey of a particular quality for different lengths of time, some buffering of the low-quality phytoplankton may occur through compensatory feeding (Meunier *et al.,* 2012), but more severe consequences for growth and fitness are expected.

Feeding on resources that do not match the consumer’s metabolic requirements substantially reduces survival, growth and reproduction of consumers (Sterner and Elser, 2002). These types of negative effects are likely strong for homeostatic consumers that do not store large amounts of nutrients, such as copepods from temperate regions (Meunier *et al.,* 2014). Consequently, many homeostatic organisms have adopted a variety of feeding behaviors, such as selective feeding, to cope with low resource quality and to minimize the metabolic costs of ingesting nutrient-poor resource (Cowles *et al.,* 1988; Jakobsen *et al.,* 2006; Jakobsen and Hansen, 1997; Meunier *et al.,* 2012; Meunier *et al.,* 2016; Meunier *et al.,* 2018; Schatz and Mccauley, 2007). However, the mechanisms underlying selectivity based on food quality, as well as the efficiency of this behavior and its influence on the consumer’s somatic growth rate, have rarely been quantified. Although copepods can differentiate between stoichiometrically distinct phytoplankton within a single species, and selectively consume more of the resource that best matches the consumer’s elemental requirements (Cowles *et al.,* 1988; Meunier *et al.,* 2016), it remains unclear whether resource quality detection is solely (and directly) based on elemental content, or whether it is driven by other algal characteristics that are linked to changes in the nutrients stoichiometry (see Meunier *et al.,* 2018).

Beside feeding behavior, zooplankton can also modulate their swimming behavior. Although copepods are well known to change their behavior depending on the concentration of food in the environment (Bochdansky and Bollens, 2004; Leising and Franks, 2002; Tiselius, 1992), responses to changes in resource quality, as characterized by stoichiometry, are less well understood. Recently, Herstoff *et al.* (2019) quantified displacement and movement patterns of different life stages of the marine copepod *Acartia tonsa* in response to different phytoplankton stoichiometries. They observed that different life stages were sensitive to different nutrient elements in phytoplankton, with movement patterns in copepodites generally varying with N content, and in adults with both N and P content. Although Herstoff *et al.* (2019) hypothesized that *A. tonsa* alters its swimming behavior in response to resource quality in ways that may allow the selective use of high-quality phytoplankton patches, this hypothesis remains untested.

Here, we studied how the presence of high-quality phytoplankton influences the growth and behavior of copepod nauplii, and structured our work around two hypotheses. First, we hypothesized that the growth of nauplii is faster when high-quality (P-rich) and low-quality (P-poor) phytoplankton are mixed rather than offered separately, as nauplii do not store nutrients and can obtain a balanced diet from a mixture through selective feeding. Second, we hypothesized that nauplii swim faster when given regular pulses of high-quality phytoplankton, as this mimics the presence of patches for which nauplii may actively search. Conversely, when high-quality and low-quality phytoplankton are mixed, it may be more advantageous for copepod nauplii to save energy by reducing swimming speed while extracting the high-quality food items from the mixture through selective feeding. Last, using the results from our experiments, and data previously published by Meunier *et al.* (2013), we assessed how the C: P ingested by nauplii is influenced by selective feeding and prey swimming speed.

## MATERIAL AND METHOD

### Algae husbandry

New batch cultures of the cryptophyte *Rhodomonas salina* were prepared daily to ensure a constant supply of food of particular qualities for the experiments. *Rhodomonas salina* cultures were grown for 3 days either in F/2 medium (Guillard and Ryther, 1962) (P-rich algae) or in F/2 medium without phosphate addition (P-poor algae). Previous work showed these two algal qualities are of high (P-rich) and low (P-poor) nutritional value for copepod nauplii (Meunier *et al.,* 2016). All cultures were grown in 1 L bottles under high light (185 μmol m^−2^ s^−1^) at a 12:12 light:dark cycle, at 18°C (for details see Meunier *et al.,* 2016). Culture densities were measured with a CASY cell counter (Schärfe System CASY Cell Counter and Analyzer System). To measure cellular C and P contents, an estimated 200 μg C were filtered on precombusted GF/F filters. Cellular C content was measured with a Vario Micro Cube elemental analyzer (Elementar, Hanau, Germany) and P was analyzed as orthophosphate after acidic oxidative hydrolysis with 5% H_2_SO_4_ (Grasshoff *et al.,* 1999). The culture treatments resulted in statistically significant differences in phytoplankton quality (Table I). Although the P-rich and P-poor prey did not differ in their C-content, P-rich prey had six times higher P-content and five times lower C:P ratio than P-poor prey (Table I).

**Table I:** Mean *R. salina* cellular carbon (C) and phosphorus (P) content (pg cell^−1^)

	P-rich prey (±std)	P-poor prey (±std)	
C [pg cell^−1^]	58.9 (8.8)^a^	47.6 (4.8)^a^
P [pg cell^−1^]	1.21 (0.11)^a^	0.20 (0.04)^b^
C:P (molar)	125.5 (15.5)^a^	617.5 (83.9)^b^

Cultures were grown to be P-rich or P-poor. Letters (a, b) indicate statistically significant differences between treatments (one-way analysis of variance, *P* < 0.05).

### Copepod husbandry

Eggs of the calanoid copepod *Acartia tonsa* were produced in 200-liter cylindrical tanks, where the animals were cultivated at 18°C at a 12:12 light:dark cycle. Copepods were fed *ad libitum* with the algae *R. salina*. Eggs were siphoned daily from the bottom of the tanks and stored in seawater at 4°C for later use. To hatch nauplii for our experiments, the stored eggs were incubated in fresh, 0.2 μm sterile filtered seawater at 18°C. Because the growth rate and feeding behavior of the copepod *A. tonsa* is known to vary widely during the course of its ontogeny (Meunier *et al.,* 2016), we minimized age differences between individuals by collecting nauplii hatched between 24 h and 36 h of incubation, during the hatching peak.

Directly after hatching, nauplii were sampled for initial C biomass by taking five samples of 200 individuals each on precombusted GF/F filters. These filters were subsequently analyzed with a Vario Micro Cube elemental analyzer (Elementar, Hanau, Germany). The remaining nauplii were transferred to 1 L Schott bottles filled with artificial seawater, with a salinity of 32, at a density of 400 individuals per liter.

### Influence of P-rich phytoplankton availability on the growth and swimming of copepod nauplii

We created two feeding treatments to test whether the relative proportions of P-rich versus P-poor phytoplankton quality available, or the amount time spent feeding on P-rich versus P-poor phytoplankton quality, influences the growth rate and swimming behavior of copepod nauplii. In both treatments, the proportion of exposure to P-rich phytoplankton was equivalent; however, nauplii were either exposed to different proportions of P-rich and P-poor food within a single phytoplankton mixture, or were exposed to different proportions of time in solely P-rich or P-poor food.

The first treatment consisted of mixtures of P-rich and P-poor phytoplankton offered in nine different duplicated proportions (Mixture Treatment, Table II). We fed each of the 18 nauplii incubations *ad libitum* by providing daily a total of 20 000 algal cells per nauplius (8000 cells.mL^−1^, 400 μgC L^−1^). Although this phytoplankton biomass likely saturated food intake by nauplii, it is well within the range of the biomass that phytoplankton blooms can reach, and therefore represents a realistic feeding scenario. Nauplii were gently sieved onto a 75 μm mesh nylon filter, and the totality of the seawater was replaced daily. The required volumes of algae for feeding were taken directly from the culture bottles and added to the containers with the nauplii.

**Table II:** Summary of the two treatments used in the experiment

Mixture treatment		Time treatment		C:P (molar)
% P-rich prey	% P-poor prey	Time with P-rich prey	Time with P-poor prey	
0%	100%	0 h (0%)	24 h (100%)	618
2%	98%	0 h 30 min (2%)	23 h 30 min (98%)	559
4%	96%	1 h (4%)	23 h (96%)	515
8%	92%	2 h (8%)	22 h (92%)	445
12.5%	87.5%	3 h (12.5%)	21 h (87.5%)	387
21%	79%	5 h (21%)	19 h (79%)	312
29%	71%	7 h (29%)	17 h (71%)	266
41%	59%	10 h (41%)	14 h (59%)	219
100%	0%	24 h (100%)	0 h (0%)	126

Nauplii were either offered different amounts of P-rich prey in within a mixture, or were offered P-rich prey for different amounts of time. In both the mixture and the time treatments, the proportion of exposure to P-rich prey was equivalent. The resulting phytoplankton C:P ratio in each treatment was calculated with the data presented in Table I. All nine of the mixture and time treatments were run in duplicate.

Using similar techniques, a second feeding treatment was created where P-rich and P-poor phytoplankton were offered separately to nauplii in nine different duplicated proportions for different amounts of time during the day (Time Treatment, Table II). These time proportions were created such that nauplii experienced the same proportions of P-rich and P-poor phytoplankton as in the food mixture treatment as described above. We ensured that each of the 18 nauplii incubations were fed *ad libitum* by providing 20 000 algal cells per nauplius of either P-rich or P-poor *R. salina*. After the required amount of time with a given food type was reached, the totality of the seawater was exchanged, and new food was given.

All incubation bottles were homogenized several times each day, to ensure a homogenous environment. After 5 days of incubation, 200 nauplii per treatment were sampled on precombusted GF/F filters. The C biomass of these filters was subsequently analyzed with a Vario Micro Cube elemental analyzer (Elementar, Hanau, Germany). These measurements were used, together with the initial C biomass data, to determine C-specific growth rates.

To measure the swimming speed of copepod nauplii, we also sampled all replicates of each treatment at the end of the5-day incubation period. We used the video imaging setup described by Herstoff *et al.* (2019) (see supplements for detailed information). Overall, the 85% of the nauplii filmed were in stage NV. In brief, we used a two-chambered Plexiglas box with interior dimensions that were 5 cm long × 2.5 cm wide × 2.5 cm deep. The back of a 90° prism was coated with a reflective mirror and placed within one side of one chamber, and the animals and food into the adjoining chamber. A Phantom MIRO LAB 110 monochrome high-speed camera (Vision Research) connected to an Olympus SZX16 stereo microscope, adjusted at a ×1.6 magnification, was positioned above the chambers. The chamber was illuminated externally with an Olympus KL 1500 LCD (color 176 temperature of 3300 K). This system is similar to that used in the double-mirror technique described by Ramcharan and Sprules (1989), and allowed us to use a single camera to simultaneously track movement in two planes, and thus removed the need to synchronize two separate video sequences in time.

### Selective feeding

We conducted a selectivity experiment to assess the efficiency with which copepod nauplii selectively ingest P-rich phytoplankton. We also tested whether feeding history influences selective feeding behavior using nauplii that had been fed 100% of the time with either P-rich or P-poor phytoplankton. Six replicates of a mixture of 50% P-poor and 50% P-rich was prepared in 50 mL cell culture flasks and diluted with artificial seawater, which contained no nutrients, to a nauplius:phytoplankton ratio of 1:20 000. These incubations were conducted following the methods of Meunier *et al.* (2016) and ran for 10 h. Previous experiments have shown that algal nutrient stoichiometry does not change during this period (Meunier *et al.,* 2012).

At the end of the 10 h incubation, samples were fixed with formalin (formaldehyde 20% buffered with hexamine) and stored in a cool, dark place. The samples were analyzed using the fluorescence method described by Meunier *et al.* (2012), and at least 300 cells of each algal quality were counted. Feeding selectivity, *α*, was calculated according to Chesson (1978, 1983). Significance of the selectivity was tested against *α* = 0.5 (Student’s *t*-test), using the different replicates of the selection experiment.

Last, we assessed the influence of selective feeding based on prey quality on the ingested C:P. We used the measured *α* to compute the naupliar diet C:P in each of the nine mixtures of P-rich and P-poor phytoplankton in the above-described experiment. To do so, we weighed the C and P contents of both phytoplankton qualities with their respective *α* for each of the nine mixtures and computed the C:P ratio. Furthermore, phytoplankton C:P influences its swimming speed, which in turn influences predator–prey encounter rates (Meunier *et al.,* 2013), and the phytoplankton detection by nauplii. Thus, we weighed phytoplankton C and P contents measured in this experiment with the algal swimming speeds measured by Meunier *et al.* (2013) to assess the influence of selective feeding based on prey swimming speed on the ingested C:P. We compared these results, on the influence of naupliar selectivity for prey quality and swimming speed, to the results expected if nauplii were completely non-selective, such that naupliar diets simply reflected the abundance of P-rich prey in the environment. To substantiate this analysis, we evaluated the frequency at which nauplii encounter P-rich and P-poor prey using the encounter equation of Gerritsen and Strickler (1977):

$$ \mathrm{CGS}=\frac{\left(\pi \mathrm{R}2\mathrm{N}\right)}{6}\ \frac{{\left(x+y\right)}^3-\mid x-y\mid 3}{xy} $$

whereby CGS is the encounter rate by a single predator in a second (prey s^−1^ predator^−1^), R is the encounter radius set to 200 μm for *A. tonsa* nauplii (Visser, 2007), N is the number of prey per cubic meter, and x and y the velocity of the prey and predator, respectively.

## RESULTS

**Fig. 1:**
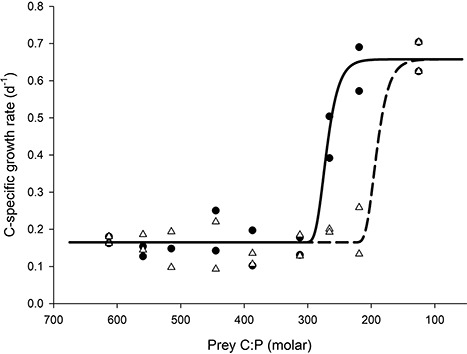
Growth rate of nauplii exposed to different mixtures of P-rich prey (white triangles and dashed regression line) or offered P-rich prey for different percentages of time (black circles and solid regression line). The lines represent logistic regressions fitted to the data.

We first tested the C-specific growth rates of copepod nauplii offered different mixtures of, or exposed for different amounts of time to, P-rich and P-poor food. The nauplii incubated for 0–20% of the time with P-rich phytoplankton, or in mixtures with 0–20% of P-rich phytoplankton (C:P between 320 and 620), both had low grow rates, ca. 0.15 d^−1^ (Fig. 1). When offered mixtures containing 20–40% P-rich phytoplankton (C:P between 220 and 320), nauplii still had low growth rate, below 0.3 d^−1^ (Fig. 1). However, incubating nauplii for >20% of the time with P-rich phytoplankton (C:P < 320) increased their C-specific growth rate, which reached a maximum of 0.65 d^−1^ for nauplii incubated over 40% of the time (C:P < 220) with P-rich phytoplankton (Fig. 1). Overall, the logistic regressions indicate that the growth reaction to increasing phytoplankton P content comes at a lower P-concentration in the time than in the mixture treatment.

**Fig. 2:**
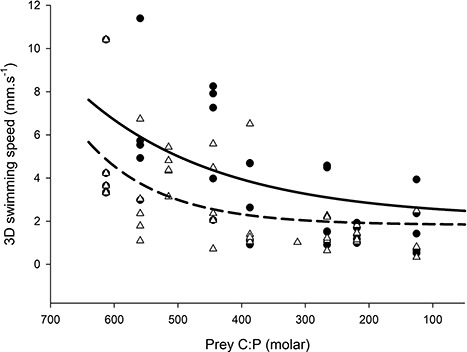
Swimming speed of nauplii that were exposed to mixtures with different amounts of P-rich prey (white triangles and dashed regression line), or were offered P-rich prey for different percentages of time (black circles and solid regression line).

Second, we considered how naupliar swimming speed was influenced by exposure to different mixtures of, and amounts of time in, P-rich and P-poor food (Fig. 2). Analysis of covariance, after testing and finding that the slopes were not significantly non-homogeneous (*F*_1,68_ = 0.3; n.s., supplementary figure), with proportion of exposure to P-rich phytoplankton as the continuous variable, the two types of treatment as the categorical variable, and log (swimming speed) as the dependent variable, showed a significant treatment effect (supplementary figure). Copepods offered P-rich prey for different amounts of time swam significantly faster than copepods offered different mixtures of P-rich prey (*F*_1,69_ = 4.55, p = 0.036). In both mixture and time treatments, naupliar swimming speed decreased with increasing proportions of P-rich phytoplankton, and reached a minimum of ca. 2 mm s^−1^ when solely offered P-rich phytoplankton (Fig. 2).

**Fig. 3:**
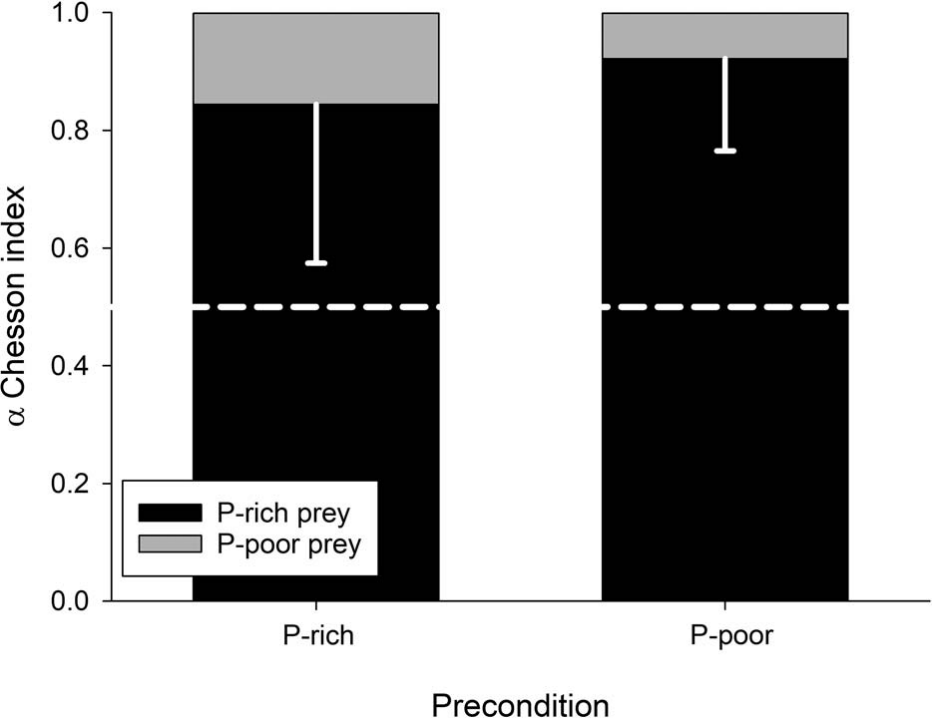
Mean selectivity α (6 replicates, -SD) of copepod nauplii offered prey that was P-rich (black bars) and P-poor (gray bars). Nauplii were either offered P-rich or P-poor food during the 5-day preconditioning period. The white dashed line at α = 0.5 indicates no selectivity.

**Fig. 4:**
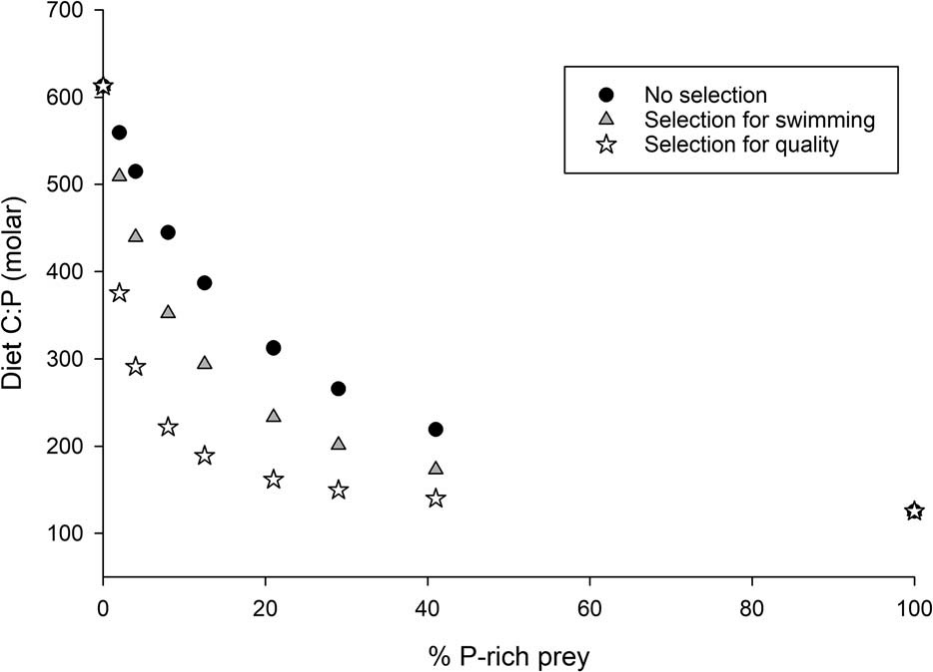
Influence of selectivity on a theoretical naupliar diet C:P. Black circles represent no selection, where the naupliar diet C:P simply reflects the proportion of P-poor and P-rich prey available within a mixture. Gray triangles represent naupliar selectivity based on prey swimming alone, with greater preference for faster swimming, P-rich prey. White stars represent naupliar selectivity based on prey quality alone, with greater preference for higher-quality, P-rich prey.

Third, we assessed the efficiency with which copepod nauplii selectively ingest P-rich phytoplankton. We observed that copepod nauplii fed selectively on P-rich phytoplankton, and that the two types of preconditioned nauplii had similar selectivity indices of α = 0.85 and 0.92 (significantly different from α = 0.5) for P-rich and P-poor preconditions, respectively (Fig. 3). Combining our data as presented above with data from the literature, we assessed how selective feeding and prey swimming speed influence the ingested C:P ratio. We observed that, since P-rich phytoplankton swim faster than P-poor phytoplankton (Meunier *et al.,* 2013), nauplii should have higher encounter rates and consequently ingest more P-rich than P-poor phytoplankton, which reduces naupliar diet C:P ratio (Fig. 4). Moreover, we observed that selective feeding for P-rich phytoplankton lowers diet C:P, and that this feeding behavior enables nauplii to obtain a diet C:P that maximizes their growth rate when at least 20% (C:P < 320) of P-rich phytoplankton is present in a mixture of different phytoplankton qualities (Fig. 4). Overall, selectivity based on prey quality seems to more strongly influence dietary C:P, as compared to no selectivity or even selectivity based on prey swimming speed alone. This result is substantiated by an analysis of the Gerritsen and Strickler encounter equation, which indicates that, in our experiment, nauplii encountered P-poor prey (0.45 prey s^−1^ nauplius^−1^) three times more often than P-rich prey (1.25 prey s^−1^ nauplius^−1^).

## DISCUSSION

Aquatic environments are not homogenous and there is evidence that phytoplankton cells of high and low nutritional value are mixed (D’ovidio *et al.,* 2010), but we still know very little about how differences in prey quality influence consumers, particularly at scales relevant to grazing organisms. We do know that many species of zooplankton have evolved mechanisms to selectively remove high-quality cells from such heterogeneous mixtures. Here, we went beyond this to assess the benefits of such behavior and we observed that copepod nauplii can adjust their diet C:P through selective feeding for P-rich phytoplankton. While our results here, together with other studies, show that resource quality influences selective feeding and ingestion rates (Cowles *et al.,* 1988; Meunier *et al.,* 2012; Meunier *et al.,* 2016; Sanders and Wickham, 1993; Zhang *et al.,* 2017), the mechanisms involved in quality recognition remain largely unknown. Nutrient limitation of phytoplankton cells can alter the biosynthesis of specific molecules (Harrison *et al.,* 1990; Shifrin and Chisholm, 1981), which may influence how consumers sense food items. For instance, dissolved nitrogen concentration was shown to influence the production of mannose located at the phytoplankton cell surface (Martel, 2009), which is involved in the biorecognition processes (Roberts *et al.,* 2011; Wootton *et al.,* 2007). Recent advances in plankton ecology indicate that prey may be perceived first mechanically, either remotely for motile prey, or with near-field perception for non-motile ones (Goncalves and Kiørboe, 2015; Kiørboe, 2016; Tiselius *et al.,* 2013). According to this body of literature, it is only after touching the prey, or having the prey very close to the copepod’s setae, that chemical sensing (gustation) could act. The importance of prey motility for copepod predation is likely important in ambush feeders like *A. tonsa* nauplii (Bruno *et al.,* 2012), in which the prey motility component (as the predator velocity is rather low) becomes very relevant (Gerritsen and Strickler, 1977). This supports our study as we have observed that, as a consequence of prey swimming speed, nauplii should encounter P-poor prey three times more often than P-rich prey at similar prey concentrations.

Because selective feeding improves naupliar diet, we hypothesized that naupliar growth would be higher when high-quality and low-quality phytoplankton are mixed rather than offered separately. Our results contradict our hypothesis. We found that, for intermediate concentrations of P-rich phytoplankton, the growth rate of nauplii was lower when high- and low-quality phytoplankton were mixed, compared to when both phytoplankton types were offered at separate times. Thus, despite the ability of copepod nauplii to selectively graze phytoplankton based on its quality, the presence of low-quality phytoplankton impaired naupliar growth. As we computed that selective feeding is an efficient behavior to improve diet C:P, this negative effect on growth suggests that selective feeding has an important energetic cost for copepod nauplii, as has been shown for a large range of consumers (e.g. Arifin and Bendell-Young, 2001 ; Porter and Mcdonough, 1984). According to the encounter equation of Gerritsen and Strickler (1977), in our experiment nauplii encountered ca. 25 P-rich or 75 P-poor prey cells per minute, while they ingested on average 4 cells per minute. This could indicate that, at the ambient prey concentrations, handling at the mouth limits food intake, rather than gut transit and digestion. Conversely, we observed that temporal segregation of P-rich and P-poor phytoplankton can be advantageous for copepod nauplii growth. This response could be attributed to the ease of feeding on one prey type at a time, whereas feeding on a mixture can be costly since the handling of different prey is then crucial. Alternatively, our results may suggest that nauplii are able to store nutrients for a short time. This may enable nauplii to cope with being deprived of high-quality phytoplankton for a few hours. This is supported by results from Calliari and Tiselius (2005) who observed that the copepod *Acartia clausi* is able to integrate the food intake over 24 h and to yield similar egg production rates, egg hatching success and gross growth efficiency when offered two types of food as a mix or shifted over time. It is likely that copepods rely not only on selective feeding as a means to handle uneven phytoplankton quality, but also that they adjust their swimming activity to make efficient use of food patches (Herstoff *et al.,* 2019).

Because copepod nauplii may actively search for, and aim to stay within, high-quality phytoplankton patches, we hypothesized that nauplii would swim faster when regularly given pulses of high-quality phytoplankton, as this mimics the presence of patches. Conversely, we predicted that when high- and low-quality phytoplankton were mixed, it may be more advantageous for copepod nauplii to save energy by reducing swimming speed to extract the high-quality food items from the mixture through selective feeding. Overall, we observed that swimming speed decreased with increasing phytoplankton P-content, a behavioral adjustment, which may improve utilization of heterogeneously distributed high-quality food in the field. While copepods are well known to change behavior depending on food concentrations (Bochdansky and Bollens, 2004; Leising and Franks, 2002; Tiselius, 1992; Tiselius *et al.,* 1993), studies of copepod behavioral responses to resource quality *in situ* are lacking, partly because measuring resource quality in the field is difficult. In this context, it is important to consider that, for the encounter rates between copepods and individual motile phytoplankton cells, the swimming velocity of the algae are important, while for the encounter rate between copepods and food patches, the performed swimming velocities of copepods are relevant. Herstoff *et al.* (2019) used high-speed videography to quantify the influence of phytoplankton quality on displacement and movement patterns of marine copepods in a laboratory setting. They observed that swimming speed was lower when copepods were offered high-quality phytoplankton and that copepods generally adjusted their behavior in response to resource quality in ways that may allow the selective use of patches of high-quality phytoplankton. In support of our hypotheses, we observed that nauplii that had been offered mixtures of high-quality phytoplankton swam more slowly, as compared to animals that had been given equivalent periods of time with high-quality phytoplankton. However, this is somewhat suprising considering that nauplii staying in a mixture of high- and low-quality phytoplankton grew more slowly than nauplii exposed to equivalent periods of time with high-quality phytoplankton. Taking these results together, we suggest that the strategy adopted by copepod nauplii is to ‘take no chances.’ Because of their high growth rate, copepod nauplii have high metabolic requirements. Moreover, *A. tonsa*’s inability to store energy means that nauplii require a constant food supply. Hence, feeding on resource of suboptimal quality probably remains a better option than leaving a phytoplankton patch and risking starvation.

Overall, we show that *A. tonsa* nauplii are sensitive to phytoplankton P content. Because the high growth rate of these consumers requires a substantial P supply, copepod nauplii have evolved a range of strategies to best locate high-quality phytoplankton and selectively extract these food items from the environment. Nevertheless, there are potential limitations to such physiological and behavioral adaptations, as we identified that the presence of low-quality phytoplankton cells impairs naupliar growth. These results suggest that deviations from optimal resource C:P ratio may cause sharp growth reductions for nauplii.

## Supplementary Material

Supplementary_figure_fbaa037

The_availability_of_phosphorus_supplements_fbaa037

The_availability_of_phosphorus_supplement_figure_fbaa037
