# Supplementary figures and images for: A matter of time and proportion: the availability of phosphorus-rich phytoplankton influences growth and behavior of copepod nauplii
Source: J Plankton Res. 2020 Aug 27;42(5):530–8. doi: 10.1093/plankt/fbaa037 (PMC7484934; doi:10.1093/plankt/fbaa037)

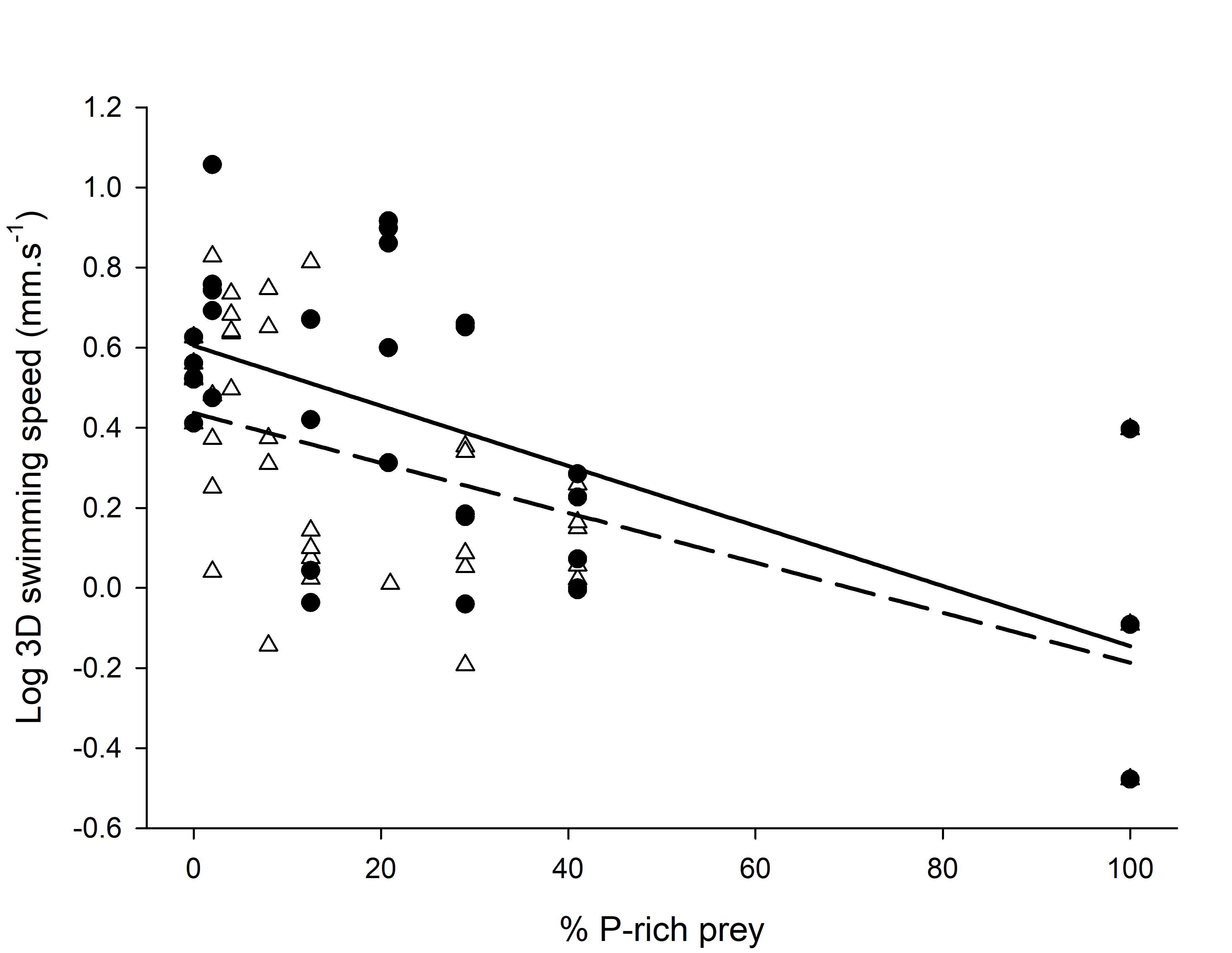

Supplement: Supplementary_figure_fbaa037 [file supplementary_figure_fbaa037.jpeg]
