# Supplementary material for: A matter of time and proportion: the availability of phosphorus-rich phytoplankton influences growth and behavior of copepod nauplii
Source: J Plankton Res. 2020 Aug 27;42(5):530–8. doi: 10.1093/plankt/fbaa037 (PMC7484934; doi:10.1093/plankt/fbaa037)
Supplement: The_availability_of_phosphorus_supplements_fbaa037 [file the_availability_of_phosphorus_supplements_fbaa037.docx]

*Video imaging*

The setup for the imaging trials was the same as described in Herstoff et al. (2019). In brief, directly before filming, nauplii were gently sieved onto a 75µm mesh nylon filter and then rinsed into 18°C artificial seawater with a salinity of 32 containing no food. To initiate the video bout, 50 nauplii were placed in the filming chamber filled with 43 mL of artificial seawater without food. This nauplii concentration falls within the range used in other copepod behavioral studies (Henriksen *et al.*, 2007, Titelman & Kiørboe, 2003). Nauplii were acclimated to lighting conditions for five minutes before imaging began. Imaging was conducted at a magnification of 16×, a video resolution of 786 × 786 pixels, an exposure time of 588 μs and a frame rate of 1,250 s^-1^. We captured 15 separate video clips for each of the two replicates from each treatment. We digitized 10 total naupliar movement tracks for each treatment, and we manually determined the X, Y and Z positions for the focal animal every 10^th^ frame. Clips were selected for digitation without knowing from which treatment individual clips were derived, and selections were based on video clarity, the length of the clip (≥ 200 frames), and whether the focal animal was clearly not touching the wall or bottom of the chamber. We used the scatterplot3d package (Ligges & Mächler, 2002) in R to create 3-d swimming tracks for each animal from which we quantified swimming speed, which was analyzed with statistical packages in R (R Core Team, 2017).

Henriksen, C. I., Saiz, E., Calbet, A. and Hansen, B. W. (2007) Feeding activity and swimming patterns of Acartia grani and Oithona davisae nauplii in the presence of motile and non-motile prey. *Marine Ecology Progress Series,* **331,** 119-129.

Herstoff, E. M., Baines, S. B., Boersma, M. and Meunier, C. L. (2019) Does prey elemental stoichiometry influence copepod movement over ontogeny? *Limnology and Oceanography*.

Ligges, U. and Mächler, M. (2002) Scatterplot3d-an r package for visualizing multivariate data. Technical Report, SFB 475: Komplexitätsreduktion in Multivariaten ….

R Core Team (2017) R: A language and environment for statistical computing. R Foundation for Statistical Computing, Vienna, Austria.

Titelman, J. and Kiørboe, T. (2003) Motility of copepod nauplii and implications for food encounter. *Marine Ecology Progress Series,* **247,** 123-135.
