# Supplementary material for: A matter of time and proportion: the availability of phosphorus-rich phytoplankton influences growth and behavior of copepod nauplii
Source: J Plankton Res. 2020 Aug 27;42(5):530–8. doi: 10.1093/plankt/fbaa037 (PMC7484934; doi:10.1093/plankt/fbaa037)
Supplement: The_availability_of_phosphorus_supplement_figure_fbaa037 [file the_availability_of_phosphorus_supplement_figure_fbaa037.docx]

Supplementary figure: Log of swimming speed of nauplii that were offered algae mixtures with different amounts of P-rich prey (white triangles and dashed regression line), or were offered P-rich prey for different percentages of time (black circles and solid regression line). An ANCOVA, with proportion of exposure to P-rich prey as the continuous variable, algae P content as the categorical variable, and log (swimming speed) as the dependent variable, showed a significant treatment effect. Copepods given P-rich phytoplankton for different percentages of time swam faster than copepods offered algae mixtures with different amounts of P-rich prey (F1,69 = 4.55, p=0.036), with non-significantly non-parallel regression lines (F1,68 = 0.3; n.s.).
